# Performance of the Novel Reactive Access-Barring Scheme for NB-IoT Systems Based on the Machine Learning Inference

**DOI:** 10.3390/s26020636

**Published:** 2026-01-17

**Authors:** Anastasia Daraseliya, Eduard Sopin, Julia Kolcheva, Vyacheslav Begishev, Konstantin Samouylov

**Affiliations:** 1 Department of Probability Theory and Cyber Security, Peoples’ Friendship University of Russia (RUDN University), 6 Miklukho-Maklaya Str., Moscow 117198, Russia; daraselia-av@rudn.ru (A.D.); sopin-es@rudn.ru (E.S.); kolcheva-yuv@rudn.ru (J.K.); samuylov-ke@rudn.ru (K.S.); 2 Federal Research Center “Computer Science and Control” of the Russian Academy of Sciences, 44-2 Vavilov Str., Moscow 119133, Russia

**Keywords:** 5G, mMTC, random access, delay, optimal resource allocation

## Abstract

Modern 5G+grade low power wide area network (LPWAN) technologies such as Narrowband Internet-of-Things (NB-IoT) operate utilizing a multi-channel slotted ALOHA algorithm at the random access phase. As a result, the random access phase in such systems is characterized by relatively low throughput and is highly sensitive to traffic fluctuations that could lead the system outside of its stable operational regime. Although theoretical results specifying the optimal transmission probability that maximizes the successful preamble transmission probability are well known, the lack of knowledge about the current offered traffic load at the BS makes the problem of maintaining the optimal throughput a challenging task. In this paper, we propose and analyze a new reactive access-barring scheme for NB+IoT systems based on machine learning (ML) techniques. Specifically, we first demonstrate that knowing the number of user equipments (UE) experiencing a collision at the BS is sufficient to make conclusions about the current offered traffic load. Then, we show that through utilizing ML-based techniques, one can safely differentiate between events in the Physical Random Access Channel (PRACH) at the base station (BS) side based on only the signal-to-noise ratio (SNR). Finally, we mathematically characterize the delay experienced under the proposed reactive access-barring technique. In our numerical results, we show that by utilizing modern neural network approaches, such as the XGBoost classifier, one can precisely differentiate between events on the PRACH channel with accuracy reaching 0.98 and then associate it with the number of user equipment (UE) competing at the random access phase. Our simulation results show that the proposed approach can keep the successful preamble transmission probability constant at approximately 0.3 in overloaded conditions, when for conventional NB-IoT access, this value is less than 0.05. The proposed scheme achieves near-optimal throughput in multi-channel ALOHA by employing dynamic traffic awareness to adjust the non-unit transmission probability. This proactive congestion control ensures a controlled and bounded delay, preventing latency from exceeding the system’s maximum load capacity.

## 1. Introduction

Nowadays, the contemporary Internet of Things (IoT) market is undergoing a rapid transformation [1,2]. Beyond the conventional applications utilizing massive machine-type communication service (mMTC) of 5G technologies, such as remote sensory equipment characterized by the asynchronous traffic demands defined in ITU-R M.2410 [3], a new generation of sophisticated services is emerging. These include critical national infrastructure applications, notably in energy grid management, forestry monitoring, and maritime logistics.

This evolution introduces a fundamental challenge: although modern 3GPP-based mMTC solutions, like Narrowband IoT (NB-IoT), successfully support extreme densities of user equipment (UE), the increasing complexity and varied demands of these new high-value services are beginning to stress the underlying wireless infrastructure [4,5]. Consequently, the air interface itself—the shared radio medium—is rapidly approaching its capacity limit and is predicted to become a bottleneck for sustaining the required performance levels [6]. This necessitates a re-evaluation of resource management and spectral efficiency within these networks.

Due to the requirements for limited power operation of end nodes, each time a packet needs to be transmitted, a new random access attempt is performed in NB-IoT systems. Since these systems utilize a multi-channel slotted ALOHA mechanism in the random access phase, their performance degrades quickly under fluctuating traffic conditions [6]. So far, a number of proactive control schemes have been proposed [7]. For example, the so-called access-barring techniques [8,9] proposed over the last decade classify UEs into several categories allocating access priorities to UEs, i.e., different probabilities for UEs to initiate the random access procedure when a packet needs to be ready for transmission. In spite of such schemes being up to standards, their performance heavily depends on the current traffic conditions, which are usually not known a priori. Furthermore, in light traffic conditions, they introduce unnecessary latency at the random access phase [10].

As an alternative approach, one may utilize reactive congestion control algorithms. Accordingly, the base station (BS) (please note that in this study, we utilize the term BS to refer to 4G/5G-grade network-side radio equipment referred to as eNodeB/gNodeB) may dynamically estimate the current load conditions and report the current access probability to UE of differing transmission attempts in the random access phase. The rationale behind this approach is that the optimal access probability for multi-channel ALOHA systems is well known. Specifically, the authors in [11] demonstrated that the optimal transmission probability is proportional to the number of UEs competing at the random access phase, *r*, as min(L/r,1), where *L* is the number of preambles. However, in the real operational system, the information about the number of active UEs competing at the random access phase is not explicitly available at either the UE or the BS. It is, however, theoretically feasible to infer it by observing the events at the PRACH channel that may provide the required information. With the development of machine learning methods, they became a suitable inference tool for that purpose.

To obtain access to the system, NB-IoT UE chooses a preamble randomly and then transmits it over a randomly selected PRACH slot. As a result of the transmission, there might be several outcomes including (i) incorrect reception due to collision with other UEs that have chosen the same preamble and PRACH slot, (ii) incorrect reception due to insufficient reception and signal strength, and (iii) correct reception of a preamble. When the system load is significant, the former type of events dominate. Despite this result having been rediscovered multiple times over the last three decades, no real system utilizes it. The rationale is that it is difficult even for BS to infer *r* dynamically by observing the events on the PRACH channel, as the inability to correctly decode a preamble may correspond to multiple events, including no preamble transmission at all, insufficient received signal strength when no collisions happened, or collisions between two or more UEs. These events are characterized by different observed powers that are also affected by various factors, with unknown distance between UEs and BS being one of the main contributors to uncertainty. Thus, given that one can (i) differentiate between the number of UEs colliding with a given preamble and (ii) provide the relation between the number of UEs colliding with a given preamble and those that had a packet ready for the transmission and (iii) feedback channel, reporting a given access probability back to the UEs, one can design a reactive access-barring scheme ensuring optimal performance of the NB-IoT system.

The idea of learning-based tuning of access class barring parameters appeared in [12], where the authors applied the self-adaptive learning property of learning automata to dynamically decide upon the access-barring (ACB) factor. Recently, with the advancement of ML techniques, the interest in access-barring schemes has been revived. The authors in [13] proposed deep reinforcement learning-based access class barring for energy-efficient operation of mMTC services. Specifically, they suggested dynamically tuning both the barring factor and the mean barring time using the past history of the system. A similar approach was proposed in [14], where the authors utilized a simpler Q-learning algorithm.

The aim of our paper is to design and assess the performance of a reactive access-barring technique for NB-IoT systems. To this aim, we first demonstrate that there is an explicit relation between the number of UEs colliding with a given preamble and the number of UEs competing for transmission at the random access phase. Then, we assess and compare the performance of ML techniques for the identification of the number of UEs colliding with a given preamble. To this end, we first utilize detailed MATLAB simulations implementing precise preambles’ waveforms and their detection mechanisms in the PRACH channel. By constructing the appropriate databases associated with different types of events in the PRACH channel, we proceed to test various ML-based classification techniques, including simple models such as tree-based classifiers (tree, random forest, and XGBoost) to more complex ones, including multi-layer perceptron and long short-term memory (LSTM) networks. Finally, we assess the delay performance of the proposed approach mathematically.

The contributions of our study are as follows:

- We proposed a new reactive access-barring technique on ML-based inference of the number of colliding UEs using SNR information only.
- We demonstrate that by utilizing ML approaches, one can detect the number of UEs that collide in a RACH channel with accuracy reaching 0.96–0.98.
- We show that utilizing the feedback of UEs using the narrowband physical downlink control channel (NPDCCH), one may improve the successful preamble reception probability and keep it consistent under overloaded system conditions.

The paper is organized as follows. We begin in Section 2 by outlining related past work. Then, in Section 3, we define our system model. There, we also establish the relation between the number of UEs colliding and the overall number of UEs attempting to access the system. In Section 4, we describe the proposed approach. The utilized simulation environment, datasets, and classification techniques for identification of the number of collided UEs is described in Section 5. Delta delivered by the proposed scheme is derived analytically in Section 6. Numerical results are then provided in Section 7. Conclusions are drawn in Section 8.

## 2. Related Work

In recent years, the problem of random access (RA) congestion in NB-IoT and LTE-M networks has attracted significant attention from the scientific community. As the number of connected devices increases, the likelihood of collisions at the access stage increases, negatively impacting network latency and throughput. One of the key mechanisms for regulating the load at the RA stage is access class barring (ACB), which allows for managing the access probabilities of different groups of UEs, thereby preventing widespread collisions.

One of the most prominent approaches in this field is the use of machine learning and adaptive control methods. In paper [15], the authors proposed a dynamic control mechanism for ACB parameters based on deep reinforcement learning (DRL) algorithms. The proposed approach improved network throughput by adapting to changing traffic conditions. Similar ideas were developed in the research [16], which implemented an adaptive access scheme that takes into account the current load and the priority of different device categories.

For high-density scenarios, significant attention is paid to reducing latency and managing traffic priorities. Papers [10,17] focus on load balancing and minimizing latency by introducing differentiated access probabilities for devices with different response time requirements.

Fundamental research, such as [8], analyzes the impact of ACB parameters on network performance under various traffic levels. These findings have led to more advanced solutions based on data analysis and machine learning, including [18], which uses predictive models to adapt access strategies in real time.

In terms of optimizing RA parameters, paper [19] offers an analytical approach to configuring RA channels to minimize the likelihood of collisions. Review article [7] highlights modern methods and key areas—from classic ACB schemes to ML-based solutions.

Earlier research in the field of machine-type communication (MTC) laid the foundation for modern solutions. In particular, ref. [20] proposed a congestion control method for MTC networks using femtocells, and [21,22] developed the concept of Contention Resolution Queues, aimed at reducing the probability of collisions. Later, distributed queuing (DQ) algorithms and load estimation were proposed, which allowed for more precise regulation of the number of active devices.

Current research extends these approaches using intelligent optimization methods. For example, ref. [19] optimizes preamble parameters and coverage levels, while ref. [23] implements uplink resource allocation using RL algorithms. Similarly, research on ML for RA channel resource management demonstrates the effectiveness of such methods in congestion prediction and adaptive access control.

In the early 1980s, during the beginning of wireless local area network standardization, researchers highlighted that the knowledge of the traffic load (that is, UEs having packets ready for transmission) can greatly help in designing random access protocols. However, the statistical signal processing methods available at that time were not powerful enough to provide detailed resolution for differentiating between waveforms containing a certain number of collided UEs.

The performed review highlights that there have been many attempts to improve the performance of NB-IoT systems under high and/or fluctuating traffic conditions. However, most of those artificially associate UEs with a certain class, and these UEs experience larger delays even when there is no significant traffic load. In general, there are no dynamic active schemes proposed so far that attempt to explicitly control the amount of traffic load imposed on the network. We proposed such a mechanism and demonstrated that it is feasible.

For convenience, the list of acronyms used throughout this paper is summarized in Table 1.

## 3. System Model

In this section, we will first specify the considered scenario. Then, we will describe the multi-channel ALOHA-based random access procedure utilized in the NB-IoT system. The notation used in this paper is provided in Table 2.

### 3.1. Considered Scenario

**Figure 1:**
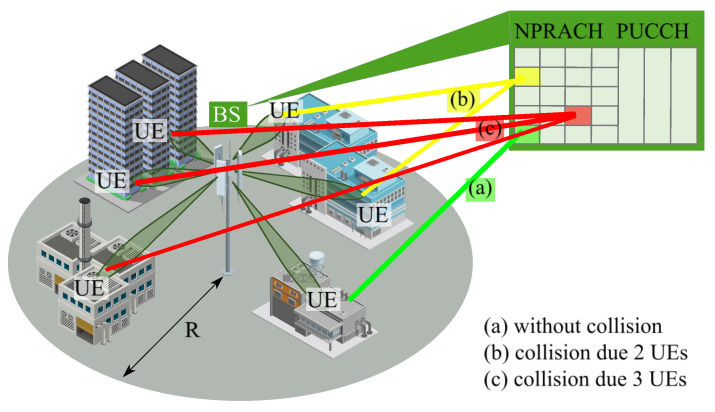
The considered mMTC deployment.

We consider the scenario shown in Figure 1, assuming a cell of circular configuration with radius *R* and a base station (BS) located in the geometrical center. User equipments (UEs) are randomly and uniformly distributed in the cell. We explicitly concentrate on the random access over the PRACH channel.

### 3.2. NB-IoT PRACH Procedure

We consider a 4-step random access scheme. As specified in the NB-IoT standard [24], the UE detects the NB-IoT carrier by measuring the power of the received synchronization signals on the downlink. During this process, the UE also achieves time and frequency synchronization and decodes the Cell ID. The synchronization information is transmitted at intervals ranging from 24 ms to 2604 ms [25].

Once synchronization is complete, the UE configures the PRACH and initiates uplink preamble transmissions based on network-defined parameters, including repetition count and transmission power. The number of repetitions can vary between 1 and 128. Each repetition follows a predefined frequency hopping pattern and is structured into four symbol groups, each comprising five symbols and a cyclic prefix, 66.67 µs or 266.7 µs, depending on the cell radius (10 km or 40 km, respectively). As a result, a complete random access attempt can last from 5.6 ms to 819.2 ms. If the preamble transmission fails, the UE will follow a retransmission timer and a retransmission limit defined by the network. Upon receiving the preamble, the BS will adjust for any time and frequency offsets and determine the timing advance (TA) for further communication. NB-IoT reserves a minimum of L=12 orthogonal preambles out of a total of 48. The data transfer phase starts with the NPDCCH sending downlink control information (DCI).

**Figure 2:**
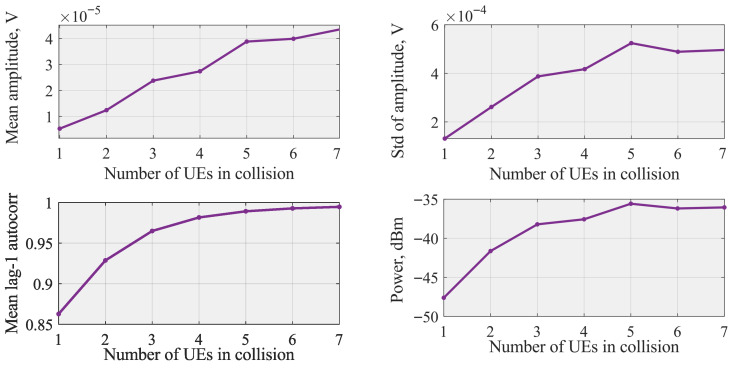
Feature values vs. number of colliding UEs. Each point corresponds to the average computed from 30,000 independent 400-sample waveform realizations.

**Figure 3:**
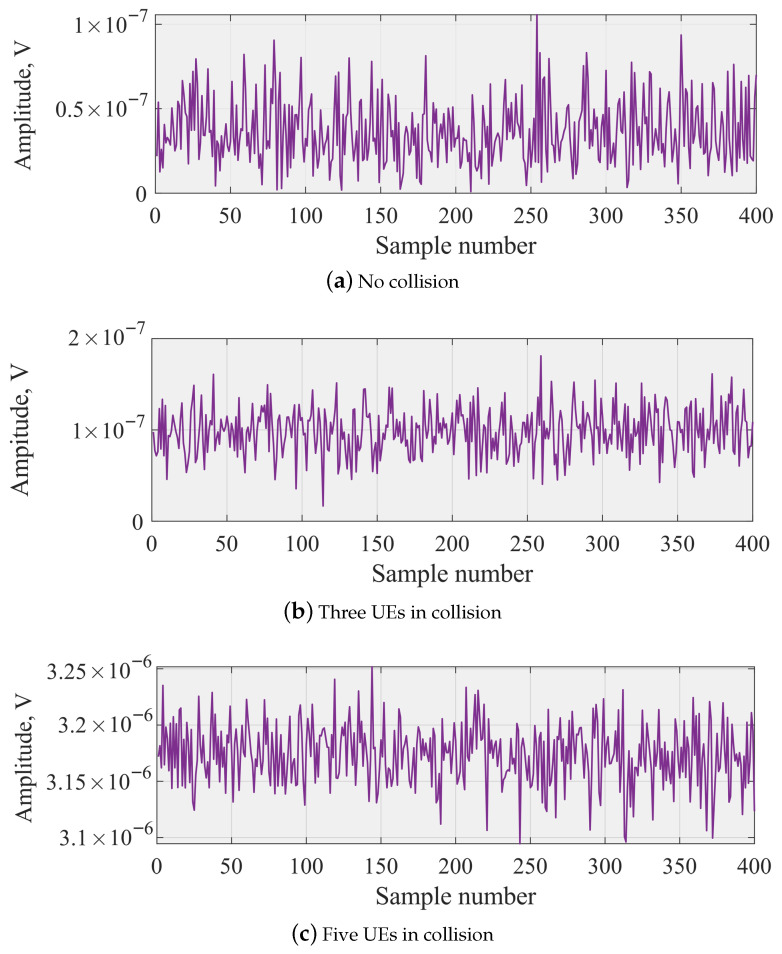
Amplitude for NPRACH with one, three, and five UEs in collision.

To obtain statistically robust metrics, all results in Figure 2 are computed using a Monte–Carlo ensemble of 30,000 waveform realizations for each number of colliding UEs. In each realization, a new NPRACH signal is generated, filtered through an independently sampled multi-path fading channel, and 400 consecutive time-domain samples are extracted. The quantities shown in Figure 3 (mean amplitude, standard deviation, lag-1 autocorrelation, and total received power) are first computed per realization and then averaged across the entire set of 30,000 realizations.

### 3.3. Propagation Model

The large-scale path loss is modeled according to the general formulation provided by Sklar [26]

(1) $$\begin{array}{c} P_{L}\left(d\right)=P_{L}\left(d_{0}\right)+10n\log_{10}\left(d/d_{0}\right)+X_{\sigma }, \end{array}$$

where *d* is the distance between the transmitter and receiver, $d_{0}$ is the close-in reference distance (assumed to be in the far-field of the antenna), *n* is the path loss exponent, and $X_{\sigma }$ is a zero-mean Gaussian random variable with standard deviation σ, representing shadow fading. In this study, we set $d_{0}=1$ m and assume $d>d_{0}$. We adopt n=3.5, which characterizes an Urban Macro-cell (UMa) environment for NB-IoT as specified in 3GPP TR 45.820 [27] and TR 36.942 [28]. For the purpose of this simulation, $X_{\sigma }$ is omitted to focus on the impact of distance-dependent path loss and multi-path fading, as the random distribution of UEs within the cell already introduces significant variability in the received signal power. The small-scale fading is modeled as a multi-path channel with four propagation paths, representing a discrete approximation of an exponential power delay profile typical for a typical urban environment. The channel impulse response includes four taps with delays of 0, 1, 2, and 3 samples and corresponding average power levels of $\left[0,-3,-6,-9\right]$ dB. Each tap is subject to independent Rayleigh fading to account for the non-line-of-sight conditions prevalent in urban areas. Finally, additive white Gaussian noise (AWGN) is added at the receiver.

### 3.4. Preamble Generation and Detection

The following PRACH channel parameters were employed for modeling: (i) *NPRACHFormat* 0 which is default format for NB-IoT PRACH, and (ii) a periodicity of 160 ms, defining the interval between available transmission instants on PRACH. The number of subcarriers is set to L=12, with zero offset. The number of repetitions is set to 1, while the UE transmits power up to 23 dBm.

For preamble detection, we assume a correlation receiver on the BS side implementing detection of transmitted preambles by comparing the received pattern with stored preambles. Preamble generation is performed individually for each equipment, and both random and forced preamble index settings for collision modeling are considered. On the BS side, the signal is correlated with the reference preamble pattern. If the maximum of the autocorrelation function exceeds a predetermined threshold (set to 50% of the ideal preamble energy), the preamble is considered detected. After detection, the number of devices that have used this preamble is analyzed, and the results are classified into the following cases: (i) successful transmission, (ii) collision due to more than one UE transmitting a given preamble, and (iii) detection failure.

## 4. The Proposed Approach

In this section, we will introduce the proposed approach. We begin with the overall description. Then, we will establish the relation between the number of colliding UEs and the number of competing UEs at the random access phase. Finally, we will specify the BS and UE side algorithms.

### 4.1. Core Idea

The core idea of the proposed approach is to estimate the number of active UEs, that is, UEs that are currently competing for resources at the NPRACH channel. This can be conducted by identifying the outcome of the NPRACH transmission. In normal conditions, NB-IoT BS just tries to decode the preamble, and if it fails to do that, it assumes that a collision has happened. We propose to take one step further and identify how many UEs collide with a given preamble. Knowing this information, one may deduce the number of active UEs based on the expression for collision probability defined below.

### 4.2. Collision Probabilities

The number of UEs that successfully passed the RA phase depends on the number of active UEs in the frame. Assume that there are currently *r* active UEs in the system competing for NPRACH resources. Let us determine the probability that a collision occurred due to a certain number of UEs. The allocations of preambles at UEs can be expressed as follows:

(2) $$\begin{array}{c} N\left(p_{i_{1}},\ldots p_{i_{s}}\right)=\frac{r!}{\left(r-ks\right)!{\left(k!\right)}^{s}}{\left(L-s\right)}^{r-ks}, \end{array}$$

where *L* is the number of preambles at the RA phase, and r!/(r−ks)! is the number of ways to select ks UEs that will fall into the specified *s* preambles, in which the first *k* UEs will go into the first preamble, the second *k* UEs into the 2nd, etc. Yet since we do not care about the order in each group of *k* UEs, we divide by k! *s* times. This can be interpreted as $r!/\left(r-ks\right)!{\left(k!\right)}^{s}$ as the number of permutations of the multiset.

Then, the number N(m) of distributions for which exactly *m* preambles contain *k* UEs is derived by applying the inclusion–exclusion principle to previously obtained (2). The upper limit of the summation is determined as $\min\left(L,\lfloor \frac{r}{k}\rfloor \right)$ from the condition that L−s≥0 and r−ks≥0. Then, N(m) is

(3) N(m)=∑s=mminL,⌊rk⌋(−1)s−msm∑1≤i1<⋯<is≤LN(pi1,…pis)==∑s=mminL,⌊rk⌋(−1)s−msmLsr!(r−ks)!(k!)sL−sr−ks,

with the probability of this event being $N\left(m\right)/L^{r}$.

According to the law of total probability, we obtain the probability that a collision will occur with exactly *k* UEs in some preambles. There are L+1 ways of having *k* UEs in 0 preambles, *k* UEs in 1 preamble, *k* UEs in 2 preambles, etc. up to *L*, respectively, which we multiply by the probability of the event that there are *k* UEs in some arbitrarily chosen preamble. Then, the probability is that the collision due to k=1,2,…, of colliding UEs is

(4) $$\begin{array}{c} P_{m,r}\left(k\right)=\sum_{m=0}^{L}\frac{m}{L}\frac{N(m)}{L^{r}}. \end{array}$$

By substituting (3) into (4) and using arithmetic transformations, (4) can be represented as follows:

(5) Pm,r(k)=∑m=0LmLr+1Lm∑s=mminL,⌊rk⌋(−1)s−mL−ms−m××r!(r−ks)!(k!)sL−sr−ks, 0≤k≤min(L,r).

**Figure 4:**
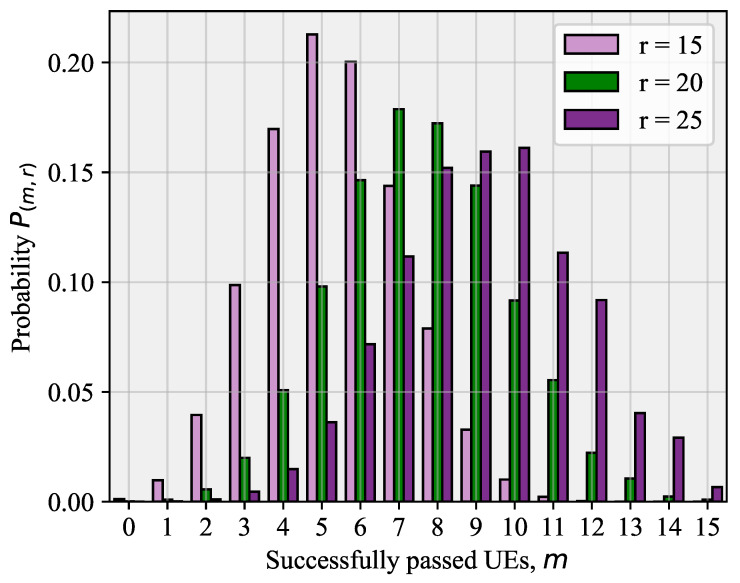
Probability $P_{m,r}\left(1\right)$ that *m* out of *r* UEs will successfully pass the RA phase.

The derived expression $P_{m,r}\left(k\right)$ in (5) establishes a direct monotonic relationship between observed preamble collisions and total offered load *r*. Specifically, higher observed collision multiplicity *k* (more UEs per preamble) corresponds uniquely to higher total competing UEs *r*. This relationship is illustrated in Figure 4 for successful transmissions (k=1), where we observe that $P_{m,r}\left(1\right)$ rapidly decreases as *r* exceeds *L*, signaling increased collision probability. Our ML classifier (Section 5) inverts this relationship: by observing collision events on PRACH, it estimates $r^{⋆}$, enabling computation of optimal access probability $p_{opt}=\min\left(L/r^{⋆},1\right)$.

For k=1, the probability $P_{m,r}\left(1\right)$ that *m* out of *r* UEs will successfully pass the RA phase is provided in [29]:

(6) Pm,r(1)=1LrLm∑i=0min(L−m,r−m)(−1)iL−mi××r!(r−m−i)!(L−m−i)r−m−i,0≤m≤min(L,r).

A particular example of (6) for L=20 is shown in Figure 4. It can be noted that the distribution becomes more symmetrical for L=r, and this in turn can be explained by the stability condition presented in [29,30].

### 4.3. BS and UE Side Algorithms

The overall procedure that is performed by every NB-IoT frame is as follows: (i) estimate the number of UE colliding at the PRACH channel, $r^{⋆}$, (ii) calculate the optimal transmission probability by utilizing $p_{opt}=\min\left(L/r^{⋆},1\right)$, and (iii) advertise $p_{opt}$ over the NPDCCH channel. The critical task is to estimate $r^{⋆}$, which is discussed in the next sections.

To illustrate the detailed operations of the proposed NB-IoT RA approach, we provide two complementary algorithms that describe the procedures at both the UE and BS sides.

The UE side procedure, presented in Algorithm 1, demonstrates how each UE participates in the RA. Specifically, it shows how UEs select preambles for transmission, monitor the feedback from the BS, and adjust their transmission probability $p_{\mathrm{opt}}$ for future frames based on the estimated number of active and colliding UEs. This algorithm captures the iterative nature of UE behavior, including handling collisions and leaving the system upon successful transmission.

The BS side procedure, shown in Algorithm 2, describes how the BS manages incoming NPRACH signals from all UEs in each frame. It includes decoding received preambles, identifying collisions, estimating the total number of active UEs $r^{⋆}$ using the collision probability model, and broadcasting the optimal transmission probability $p_{\mathrm{opt}}$ to all UEs through NPDCCH. Additionally, the BS sends Msg2 to UEs whose transmissions were successfully received. This algorithm provides a complete view of the BS operations and its coordination role in controlling UE access to shared resources.

Together, these algorithms present a detailed and practical view of the interaction between UEs and the BS in every NB-IoT frame. They bridge the theoretical analysis of collision probabilities and active UE estimation with the operational steps required to implement the proposed approach. By following these algorithms, the overall system efficiently adapts the UE transmission probabilities and manages collisions, ensuring improved performance in the RA phase.   
**Algorithm 1:** UE side operation
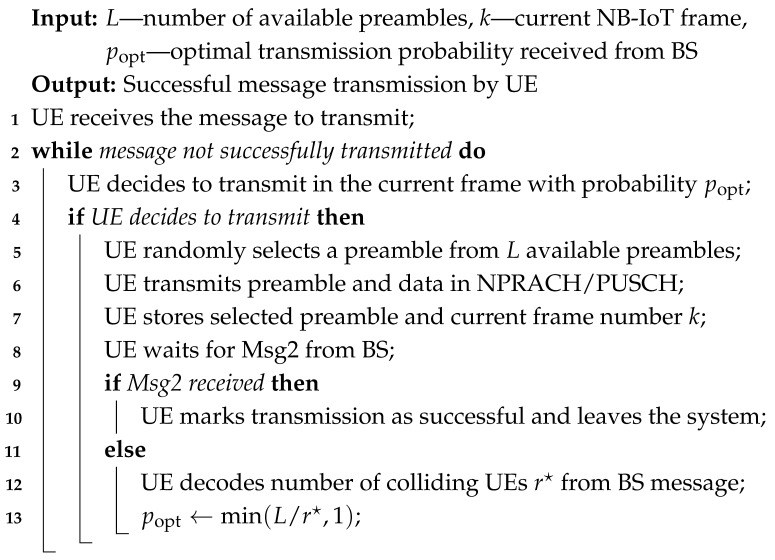


**Algorithm 2:** BS side operation

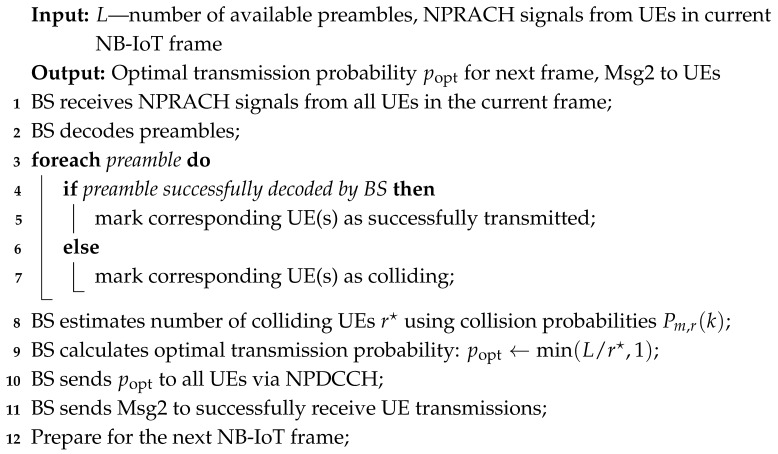



## 5. Collision Estimation Procedure

In this section, we first describe the simulation environment and data generation procedure. Then, we will introduce the considered ML algorithms. Finally, the training and testing datasets and metrics of interest are introduced.

### 5.1. Dataset Generation

We carry out our simulation campaign of the NPRACH procedure in MATLAB environment using an extension of the LTE Toolbox for NB-IoT. We utilize a standalone deployment option for NB-IoT. Attention is paid to the physical layer (PHY) signal transmission at the NPRACH channel when one or more active UEs pass the random access phase. Simulation modeling has been carried out in the MATLAB R2024b environment, using built-in LTE Toolbox functions, including *lteNPRACH*, designed to generate NB-IoT PRACH signals according to 3GPP TS 36.211. The model covers time slot generation, signal propagation, special effects including fading, thermal noise, and signal processing at the BS.

For each transmission in the random access scenario, a time series of samples of the received signal on the BS side is formed, represented as a sequence of complex numbers: $\left(s_{1},s_{2},\ldots ,s_{N}\right)$, where N=1000 is a fixed number of samples in the time domain. Each sample reflects the amplitude value of the complex radio signal received at evenly spaced moments in time during a single preamble transmission. These time series reflect the amplitude and phase structure of the signal at the receiving end and carry information about the presence of collisions (overlapping preambles from different UEs). These data are collected for each transmission simulation and subsequently used as input features to ML algorithms to automatically detect collisions and recognize the channel state based on the received signal waveform.

In order to construct the dataset, the simulation is carried out according to the following. In each simulation run, we model transmission of *j*, j=1,2,…,M UEs. The maximum coverage of was set to R=500 m. The distances to UEs were chosen to follow a uniform distribution in the coverage area BS with probability density function (pdf) $f\left(x\right)=2x/R^{2}$, 0<x<R. For each number of UEs, we performed 1000 runs each time, generating *N* time samples $s_{i}$, i=1,2,…,N. To account for differences in preamble structure, we forced the cycled use of preambles, where in the first run, the first preamble was chosen, in the second run, the second preamble was chosen, etc. When the number of UEs is greater than one, we force all UEs to choose the same preamble. These data are used as features for training models based on machine learning algorithms.

The measured amplitude time-series one, three, and five UEs choosing the same preamble are shown in Figure 3. Observe that these time-series do not contain any evident artefacts or features that can be easily utilized to make decisions upon a number of collision UEs. However, with the increase in the number of UEs choosing the same preamble, the mean amplitude increases signaling a possibility for differentiating between the number of colliding UEs using even simple classifiers such as conventional trees or random forests.

### 5.2. Utilized ML Techniques

Observe that due to the system behavior, the more EDs found themselves in a collision, the higher the power that is received at the BS in a dedicated RACH slot. Thus, in principle, the task could be solved using simple classification techniques as it does not require determining sophisticated patterns in time-series. Thus, in what follows, we will consider several algorithms having different degrees of training complexity. Those having low complexity include two of the most popular decision tree (DT) algorithms, XGBoost [31], and random forest (RF). DT and RF operate using features extracted from the data and may miss patterns that be present in the time-series. Therefore, as an alternative, we will consider long short-term memory (LSTM) networks, which capture the spatial structure of the data. We note that the proposed approach does not stipulate a certain choice of the classifier and more advanced ones such as those based on convolutional neural networks (CNN) or even Transformers can be utilized.

Given the recognized effectiveness of decision tree-based algorithms for such tasks, our focus was on these methods. We prioritized simplicity, leading us to employ the random forest technique. For comparison, we also included XGBoost, renowned as a highly powerful tree classifier, and LSTM, which serves as an example of a more complex approach.

The random forest and XGBoost models were configured with 300 trees, each having a depth of 20 branches. This specific parameter set was empirically determined to yield optimal performance. The LSTM network required 10 training epochs to achieve a comparable result to XGBoost. Notably, even this limited number of epochs demanded substantial computational time. In contrast, XGBoost attained an equivalent level of accuracy significantly faster. Consequently, XGBoost was selected for the final evaluation, as it represents a parsimonious yet highly effective and computationally efficient solution.

#### 5.2.1. XGBoost

XGBoost is currently one of the strongest machine learning algorithms used for classification and regression tasks. It represents the construction of decision trees with gradient boosting. The scheme of its operation looks like this: the first decision tree is built, which may contain a large error in calculations, after which a new tree is built, which should minimise the error of the previous one. Then, a new tree is built again, which aims to correct the errors of all existing trees. The process continues until we minimise the resulting error. The final result will be the sum of the predictions of all trees.

#### 5.2.2. Random Forest

Random forest is one of the machine learning methods that can improve accuracy and significantly reduce the probability of model overfitting. The algorithm creates several subsamples from the training data using the bootstrap method. For each subsample, a separate decision tree is built, and they can have different structures and parameters, as each tree is trained on its own subsample of data. In addition, the trees are not pruned, unlike the method with a single decision tree. When solving a classification problem, the final result is usually the one for which the trees received the most votes. Also, the final result can be calculated through the median: $Y=M(Y_{1},Y_{2},\ldots ,Y_{i})$, where $Y_{i}$ is the solution of the i-th tree. In this way, the strong influence of noise can be avoided, and the sensitivity to outliers can be reduced. Due to its high accuracy and ability to handle outliers well, random forest remains one of the most sought-after machine learning methods.

#### 5.2.3. LSTM Neural Networks

LSTM is a special type of recurrent neural network (RNN) designed to handle sequential data and solve the vanishing gradient problem that often occurs in conventional RNNs. They have been designed to handle and predict sequences, that is, data that is time-dependent. The main point of its operation is as follows: LSTM can remove and write information to the cell state using special structures called gateways. There are three main gates that help to control the cell state. The first layer is the forget gate layer. It is a sigmoidal layer that determines what information can be removed from the cell state. The next step is to update the cell state. This layer consists of two parts: the input gates layer, which determines which values need to be updated, and the tanh layer, that builds new values to replace the old ones with. In the final step, we implement all the above actions; that is, we change the state of the cell, forgetting unnecessary values and updating the data. Most of the outstanding results that have brought RNNs to mankind have been achieved thanks to LSTM, as this neural network makes inferences considering all previous events. These are networks that contain feedbacks and allow information to be stored for future work.

### 5.3. Tasks, Features, and Metrics

Formally, the goal of the introduced ML techniques is to perform multi-class classification of the received NPRACH waveforms. For the LSTM network, the input data are the time-series obtained in simulations. However, for XGBoost and DT, we define the following features that are extracted from the time-series data: (i) mean, (ii) standard deviation, (iii) values of the autocorrelation function at lag-1, and (iv) summed periodogram. The latter is defined via the sum of short-term Fourier transform (STFT)

(7) $$\begin{array}{c} S\left(m,\omega \right)=\sum_{n=0}^{W}f\left[m+n\right]w\left[n\right]e^{-j\omega n}, \end{array}$$

where S(m,ω) represents the STFT magnitude at time *m* and the frequency ω, f[m+n] is the (m+n)-th sample of the time window (0,T), and w[n] is the *n*-th sample of the window function.

Figure 2 illustrates the behavior of the selected features for classification averaged over time and experiments. Each data point shown in Figure 2 represents an average over 30,000 independent simulation realizations, and the corresponding standard deviation is negligible relative to the mean value, and therefore omitted in the visualization for clarity. As one may observe, all the selected features have strictly increasing behavior as the number of UEs colliding increases. While the trend was expected for mean and standard deviation based on the data presented in Figure 3, even summed periodogram and lag-1 NACF values increase. Another important observation is that starting from approximately five UEs choosing the same preamble, statistical properties do not vary much. This implies that simple classification techniques, such as trees and random forest, may struggle to distinguish between considered cases. To this end, we also utilize the LSTM that works directly with the time series illustrated in Figure 3, and attempt to find hidden behavior.

To evaluate the performance of the proposed algorithms, we utilize two metrics: (i) accuracy and (ii) F1. The former metric measures the proportion of correctly classified examples out of the entire dataset and is calculated as $A=N_{C}/N$, where $N_{C}$ is the number of correctly classified examples in all the categories, and *N* is the total number of examples. The F1 score combines precision and recall into a single measure by calculating their harmonic mean. This balances the impact of these metrics on the model’s quality assessment, making the F1 score useful when both performance aspects are important. We estimated the F1 for each class *k* as

(8) $$F_{1_{k}}=\frac{2P_{k}\;R_{k}}{P_{k}+R_{k}},$$

where precision for class *k*, $P_{k}$, and recall for class *k*, and $R_{i}$, are calculated as

(9) $$P_{k}=\frac{T_{P_{k}}}{T_{P_{k}}+F_{P_{k}}},\;R_{k}=\frac{T_{P_{k}}}{T_{P_{k}}+F_{P_{k}}},$$

where $T_{P_{k}}$ is the number of true positive predictions for class *k*, $F_{P_{k}}$ is the number of false positive predictions for class *k*, and $F_{N}$ is the number of false negative predictions.

## 6. Delay Assessment

In this section, we will evaluate the random access phase delay associated with the proposed approach. To this aim, consider first the Markov chain $R_{n}$ describing the number of active UEs at the RA phase. Since $R_{n}$ is non-reversible, we aggregate all states with a number of active UEs greater than a threshold *R* in one absorbing state ω. As a result, $R_{n}$ becomes an absorbing Markov chain, and its transition probability matrix $P^{\left(R\right)}$ can be represented as

(10) $$P^{\left(R\right)}=\left(\begin{array}{cc} P & a\\ 0 & 1 \end{array}\right),$$

where P is the (R+1)×(R+1) matrix that reflects the transitions between transient states, a is the exit vector to the absorbing state, and 0 is the row-vector of zeros.

**Figure 5:**
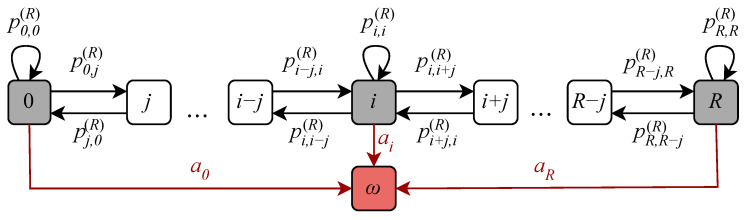
State transition diagram of the RA phase model.

The state transition diagram of the considered Markov chain is illustrated in Figure 5. Observe that there are two types of events that cause a state transition at the RA phase. We summarize them below, together with the corresponding probabilities, assuming that the system is initially in state *i* and in the next frame it moves to state *j*, i.e., $p_{i,j}^{\left(R\right)}=P\left\{R_{n+1}=j|R_{n}=i\right\}$. Note that these events can occur separately or simultaneously. Events may occur as follows:

1. *Arrival of new UEs.* If (j−i) new UEs arrive when there are *i* UEs at the RA phase, then there will be *j* UEs in total in the system.
2. *Successful UEs allocation to preambles and UEs redirection to DT phase.* In this case, the (i−j) out of *i* UEs will successfully pass the RA phase.

If both events happen simultaneously, then *k* UEs are successfully allocated to preambles and redirected to the DT phase, and thus (j−i+k) new UEs enter the system.

The probability $\delta_{i}\left(j\right)$ that *i* out of *j* active UEs, that are ready to transmit, will transmit, has a binomial distribution:

(11) δi(j)=jiπ(j)j−i1−π(j)i,

where π(j) is the transmission probability with *j* UEs ready to transmit.

Utilizing the abovementioned observations, the transition probabilities between the transient states are as follows:

(12) $$\begin{array}{c} p_{i,j}^{\left(R\right)}=\sum_{k=\max(0,i-j)}^{i}\beta_{k}\left(i\right)\alpha_{j-i+k},\;i\leq L,\\ p_{i,j}^{\left(R\right)}=\sum_{k=\max(0,i-j)}^{i}\sum_{m=\max(0,i-j)}^{k}\delta_{k}\left(i\right)\beta_{m}\left(k\right)\alpha_{j-i+m},\;i>L, \end{array}$$

for 0≤i≤R, 0≤j≤R.

Since $P^{\left(R\right)}$ is a stochastic matrix, the elements of exit vector a complement the row sums to one as follows:

(13) $$\begin{array}{c} a=(I-P)1, \end{array}$$

where I is the unit matrix and 1 is a row-vector of ones.

Let $R_{n}$ start from an empty state with no active UEs. Then, the initial vector is $e_{0}=\left(1,0,0,\ldots ,0\right)$. According to the theory of absorbing Markov chains, the average number of steps before absorption $E\tau_{\Delta }$ is given by [32]

(14) $$\begin{array}{c} E\tau_{\Delta }=e_{0}N1, \end{array}$$

where N is the fundamental matrix, $N={\left(I-P\right)}^{-1}$.

Finally, multiplying (14) by Δ, we obtain the average time before absorption, i.e.,

(15) $$\begin{array}{c} E\tau =e_{0}N1\Delta . \end{array}$$

By utilizing (15), we can make the guided choice of the threshold *R*, allowing us to decrease the computational complexity of the model. To this end, Figure 5 shows the mean time before absorption E[τ] for the system with L=100 preambles as a function of *R* for different values of arrival rates. As one can observe, R=2L=200 E[τ] approaches a constant. This effect can be interpreted such that after reaching R=2L UEs, the number of active UEs at the random access phase almost surely goes to infinity in a very short time period. Thus, choosing *R* twice, the number of preambles is sufficient.

The fundamental matrix *N* has a probabilistic interpretation [32]. Specifically, its elements $n_{ij}$ represent the mean number of visits to state *j* if the initial state was *i*. This fact can be used to obtain the transient probabilities $q_{i}^{\left(R\right)}$, 0≤i≤R as

(16) $$\begin{array}{c} q_{i}^{\left(R\right)}=\frac{n_{0i}}{E\tau_{\Delta }} \end{array}$$

To obtain the mean delay of packets in the system, we utilize Little’s law, and can see that the mean number of UEs at the RA phase:

(17) $$\begin{array}{c} \bar{R}=\sum_{i=1}^{R}iq_{i}^{\left(R\right)}, \end{array}$$

Then, the mean delay is immediately given by

(18) $$\begin{array}{c} w=\frac{\bar{R}}{\lambda_{r}}. \end{array}$$

## 7. Numerical Results

In this section, we present our results. We begin with statistical data analysis of the obtained waveforms and defined features. Then, we will proceed with the assessment of accuracy and F1 metrics for single-class and multi-class classifications of collisions on the NPRACH channel.

### 7.1. Classification Performance

**Figure 6:**
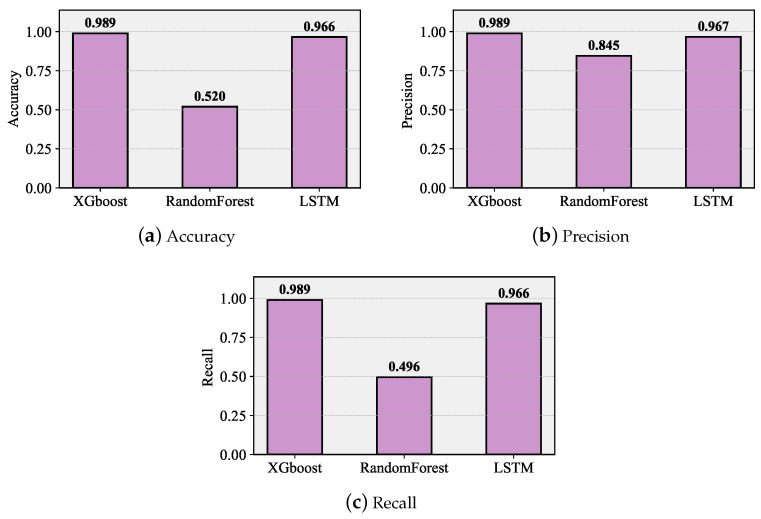
Accuracy, precision, and recall metrics for classifying collisions with {1,2,…,5} UEs at the PRACH channel.

We begin reporting our classification results by observing the accuracy, precision, and recall metrics illustrated in Figure 6 for classifying collisions with {1,2,…,5} UEs at the PRACH channel, where 1 implies that the preamble has been successfully received. Observe that accuracy is determined for overall classification, while recall and precision are specified for each class. Observing the data shown in Figure 6a, we see that out of the considered techniques, XGBoost provides the best accuracy of around 0.98, with LSTM lagging slightly behind, providing an accuracy of 0.96. The worst accuracy is observed for the random forest classifier, barely reaching 0.5. Precision and recall, in Figure 6b,c, show that the latter fails mainly in determining the successful preamble reception probability, while the other two algorithms operate almost perfectly, not allowing for misinterpreting classes. Since LTSM is generally associated with a complex learning phase as compared to XGBoost, the latter is used in what follows.

**Figure 7:**
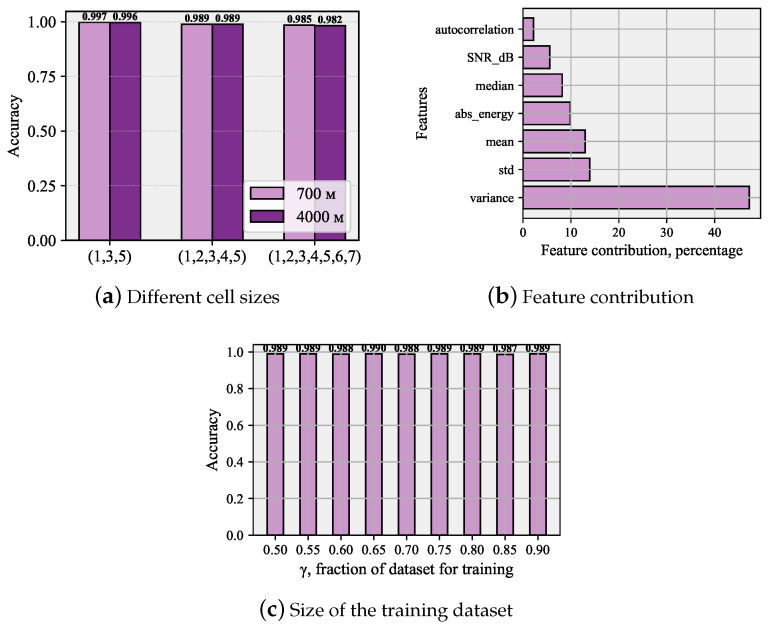
Classification performance for other systems and XGBoost classifier parameters.

To demonstrate the versatility of our approach, we now report the accuracy for two different NB-IoT BS coverage areas of 700 and 4000 m in Figure 7a, investigate the dependence of the accuracy of the learning data size in Figure 7c, and classify the feature contribution into the decision-making of XGBoost in Figure 7b. For the comparative analysis of classification outcomes across varying data split proportions, the XGBoost model was chosen based on its demonstrated optimal performance. The provided graph illustrates the full range of division ratios we tested and the resulting metrics. It is important to note that this work did not utilize cross-validation; instead, a class-stratification parameter was applied. This method ensured that the test set maintained an equal number of samples from every class. First of all, we observe that the proposed classification works well for both considered call sizes, providing comparable high accuracy. Secondly, we see that the classification can also be extended to extremely overloaded systems, correctly classifying up to seven simultaneous preamble transmissions in the same PRACH resources with a slight degradation in the accuracy metric. Furthermore, the accuracy barely depends on the learning dataset size as indicated in Figure 7c. Finally, in Figure 7b, we observe the largest weight in the decision of XFBoost classifier is introduced by the variance and mean of the waveform. Figure 7b shows that the largest contribution is made by the variance. The significant gap between the variance and standard deviation can be explained by the operation of tree-based classifiers. Variance provides more informative feature combinations for separation when using tree models.

We specifically note that experiments were carried out using Matlab simulator, UEs locations were generated using uniform distribution in the BS coverage area which is aligned with ITU-R M.2410 requirements, and no additional features were introduced that might artificially improve classification performance.

### 7.2. NB-IoT System Performance

In this section, we will illustrate NB-IoT system’s performance operating using the proposed approach and compare it to that of conventional operation. To this end, we utilized the MATLAB modeling tool to simulate these two operational regimes. We utilize a standalone deployment option for NB-IoT. Here, UEs in the coverage area of the NB-IoT BS have been uniformly distributed. To ensure true spatial uniformity of users over the cell area (i.e., without radial bias), the UEs are distributed using inverse-transform sampling in polar coordinates. Two independent random variables $U_{1},U_{2}∼\mathrm{Uniform}\left(0,1\right)$ are generated for each UE, and the spatial coordinates (x,y) are obtained as

(19) $$\begin{array}{c} r=R\sqrt{U_{1}},\;\theta =2\pi U_{2},\;x=r\cos\theta ,\;y=r\sin\theta , \end{array}$$

where *R* is the cell radius. This sampling guarantees a constant density of UEs over the entire disc area $A=\pi R^{2}$, fully consistent with the scenario assumptions of ITU-R M.2412. The time interval between arrivals E[X] follows exponential distribution resulting in the Poisson arrival process. All UEs were completely decentralized. We embedded our algorithm at the BS side, and it detects the number of collisions happening in the preceding frame at the PRACH by utilizing the XGBoost algorithm and advertises this number as a part of the DCI message in the PDCCH. UEs then estimate the optimal transmission probability by utilizing (6) and compete for PRACH resources in the next frame with probability $p_{opt}=\min\left(L/r^{⋆},1\right)$, where *L* is the known number of preambles, and $r^{⋆}$ is the number of UEs competing at the random access phase. The rest of the parameters followed the scenario defined as ITU-R M.2412.

**Figure 8:**
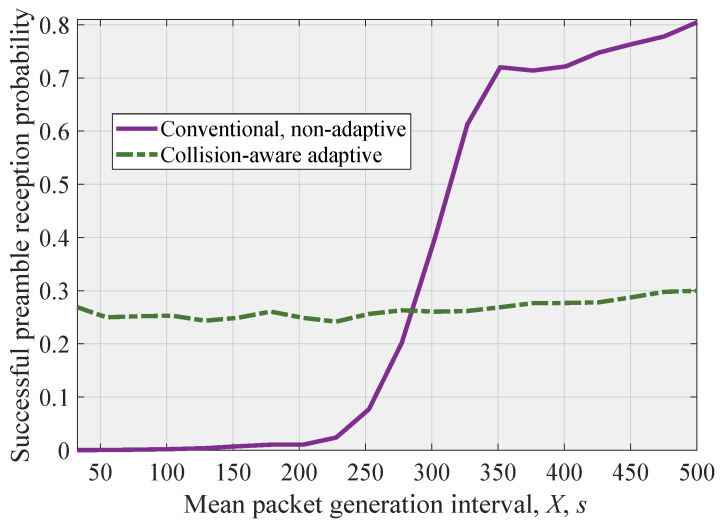
Successful preamble reception probability for considered modes.

The successful preamble reception probability as a function of the time interval between arrivals at a single UE is shown in Figure 8. As one may observe, the conventional NB-IoT RAVH procedure results in extremely low successful preamble reception probability up until E[X]=270 s, where it starts to increase. Contrarily, we observe that the proposed approach allows us to maintain a consistent successful preamble reception probability across this region.

As one may observe, up until the moment around 270 s, the conventional scheme outperforms the proposed one. Therefore, one of the extensions of the proposed approach would be to track the delay experienced by UEs in the system and to switch to the proposed scheme only when it starts to increase sharply, indicating the potential overload conditions. To implement this regime, BS in addition to monitoring and estimating the number of EDs colliding should also track the current successful preamble reception probability. This information can be available using the proposed classificator as it returns the approximate number of UEs competing for transmission.

### 7.3. Delay Performance

**Figure 9:**
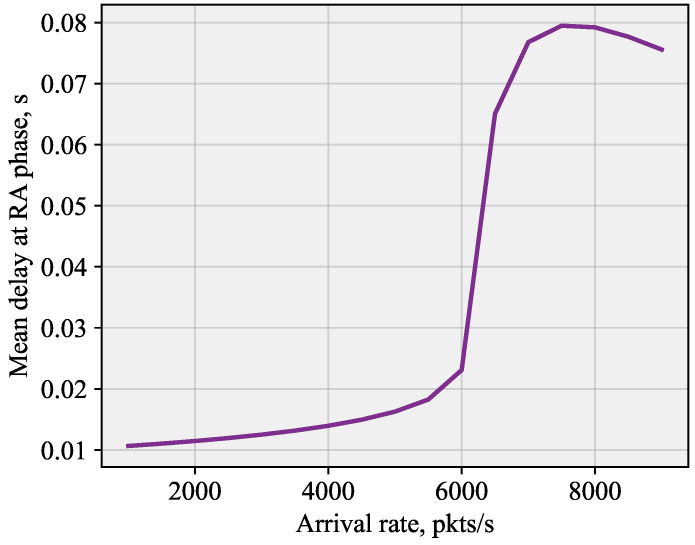
Delay performance under the proposed scheme.

In this section, we will report on the delay performance of the system that utilizes the proposed scheme. To this end, Figure 9 shows the delay as a function of the arrival rate of packets. As one may observe, the overall curve is reminiscent of that reported in Figure 8 for the optimal transmission probability. Here, we see that the delay remains low for low arrival rate. This is a region where the system is not overloaded, and the transmission probability is not affected by the BS. Once the system starts to enter the overloaded regime, which happens quite quickly, the proposed system based on the identification of the number of UEs experiencing collisions comes into play. We see that it allows the delay to rise till 8 ms and then stabilizes it at this value.

## 8. Conclusions

Nowadays, mMTC technologies providing coverage to numerous sensing devices are becoming more and more critical for various services, including remote monitoring and control, state update applications, etc. In such systems, the random access phase, generally implemented using multi-channel ALOHA algorithm, is the major bottleneck. It is known that this algorithm is characterized by the optimal number of UEs competing for access which maximizes the capacity of the system. Knowing the number of UEs competing for access is thus critical for optimizing the performance of the random access phase in mMTC technologies.

In this paper, we propose a novel dynamic access-barring technique based on the estimation of the number of UEs competing for resources at the preamble transmission phase. Specifically, by utilizing ML techniques, we proposed to deduce the current number of active UEs at the random access phase by observing the waveform of the NPRACH channel. Then, having the analytical expression providing the collision probability for the multi-channel ALOHA mechanism, we probabilistically estimated the number of UEs competing at the random access phase. This information is dynamically returned to the UEs competing for access.

Our numerical results illustrate that the proposed scheme can dynamically track the offered traffic load. Furthermore, when this load increases to a certain level, the scheme induces a non-unit transmission probability. This specific level is optimal for achieving the maximum performance of the multi-channel ALOHA scheme. The delay in the proposed scheme is kept controlled at the level corresponding to the maximum load that can be handled by the multi-channel ALOHA system.

Our future work includes testing of the proposed approach in a laboratory environment using operational NB-IoT BS and UEs. In our current setup environment, different metallic boxes and attenuation lines allow for modeling various propagation environments that may affect the core of the proposed approach—classification performance. Our aims here also include practical aspects related to modifying PDCCH signalling for informing UEs about the optimal probability for competing for RACH resources, and adaptation of the proposed schemes to different frame markups defined by NB-IoT standards. Also, we plan to test whether the algorithm could be applied in real time and provide additional insights on its online implementation, including the optimal size of the training sample.

## Figures and Tables

**Table 1:** List of acronyms.

Acronyms	Description
ACB	Access class barring
AWGN	Additive white Gaussian noise
BS	Base station
DRL	Deep reinforcement learning
DT	Decision tree
DT	Data transmition phase
DQ	Distributed queuing
IoT	Internet of things
ITU-R	International telecommunication union radiocommunication sector
LTE	Long-term evolution
LSTM	Long short-term memory
ML	Machine learning
MTC	Machine-type communication
mMTC	Massive machine-type communication service
NACF	Normalized autocorrelation function
NB-IoT	Narrowband IoT
NPDCCH	Narrowband physical downlink control channel
NPRACH	Narrowband PRACH
pdf	Probability density function
PHY	Physical layer
RNN	Recurrent neural network
PRACH	Physical random access channel
RA	Random access
RF	Random forest
SNR	Signal-to-noise ratio
STFT	Short-term Fourier transform
TA	Timing advance
TR	Technical reports
TS	Technical specifications
UE	User equipment
3GPP	The 3rd generation partnership project

**Table 2:** Notation used in the paper.

Notation	Description
$P_{L}\left(d\right)$	Path loss
$P_{L}\left(d_{0}\right)$	Constant cut-off attenuation
*L*	Number of preambles in a frame
*r*	Number of UEs competing at the RA phase
$N(p_{i_{1}},\ldots p_{i_{s}})$	Allocations of preambles at UEs
N(m)	Number of distributions for which exactly *m* preambles contain *k* UEs
$P_{m,r}\left(k\right)$	Probability that a collision will occur with exactly *k* UEs in some preambles
$p_{opt}$	Optimal transmission probability
*N*	Fixed number of samples in the time domain
*R*	Maximum coverage
f(x)	pdf of distances to UEs were chosen to follow a uniform distribution in the coverage area BS
S(m,ω)	STFT magnitude at time *m* and the frequency ω
f[m+n]	(m+n)-th sample of the time window (0,T)
w[n]	*n*-th sample of the window function
*A*	Accuracy
$N_{C}$	Number of correctly classified examples in all the categories
*N*	Total number of examples
$F_{1_{k}}$	F1 for each class *k*
$P_{k}$	Precision for class *k*
$R_{i}$	Recall for class *k*
$T_{P_{k}}$	Number of true positive predictions for class *k*
$F_{P_{k}}$	Number of false positive predictions for class *k*
$F_{N}$	Number of false negative predictions
$R_{n}$	Markov chain for the number of EDs at the RA phase
*R*	Threshold of number of active UEs
ω	Absorbing state
$P^{\left(R\right)}$	Transition probability matrix of $R_{n}$
$q_{i}^{\left(R\right)}$	Stationary probability pmf
$P^{\left(R\right)}$	Transition probability matrix
N, I	Fundamental/unit matrix
a, $e_{0}$	Exit/initial vector
$q_{i}^{\left(R\right)}$	Transient probability pmf
$\delta_{i}\left(j\right)$	Probability that *i* out of *j* active UEs, that are ready to transmit, will transmit
π(j)	Transmission probability with *j* UEs ready to transmit
$\bar{R}$	Mean number of UEs at the RA phase
*w*	Mean delay of packets

## Data Availability

Data are contained within the article.
